# Benign food-borne type B botulism presenting as unilateral internal ophthalmoplegia: a case report

**DOI:** 10.1186/s12883-022-02939-0

**Published:** 2022-11-28

**Authors:** Francesco Crescenzo, Raffaele Del Colle, Domenico Ajena, Matteo Stecca, Laura Ferigo, Francesca Rossi, Michelangelo Turazzini

**Affiliations:** Neurology Unit – Mater Salutis Hospital, Azienda ULSS 9 Scaligera, Verona, Italy

**Keywords:** Botulism, Diagnosis, Case report, Autonomic disorder, Mydriasis, Sympathetic skin response

## Abstract

**Background:**

Food-borne botulism is a rare neuromuscular junction disorder due to the effect of toxins released from Clostridium botulinum ingested by eating improperly stored food. Its classic manifestation is a rapidly evolving descending symmetrical flaccid paralysis with dysautonomia.

**Case presentation:**

We have described a case of type B food-borne botulism with a benign clinical course characterized by an initially unilateral tonic mydriatic pupil. An extensive neurophysiological evaluation inclusive of pilocarpine eye drop(s) test, facial and limbs nerve stimulation and sudomotor tests, was decisively leading the diagnostic process.

**Conclusions:**

The importance of what has been described here lies in underlining that it is always advisable to consider food-borne botulinum intoxication, even in the case of unilateral/asymmetrical internal ophthalmoplegia without generalized progressive involvement of the voluntary muscles.

## Background

Botulism is a life-threatening disease caused by the toxins produced by the spore-forming anaerobic bacterium Clostridium botulinum that inhibit presynaptic acetylcholine (ACh) release at both voluntary motor and autonomic cholinergic neuromuscular junction. There are seven distinct serotypes of C. botulinum neurotoxins, labelled A to G (BoNTs/A-G). Among them, the toxins A, B and E are the leading cause of human disease. In Italy, the incidence of botulism rates 0.02–0.04 cases per 100,000 inhabitants, of which the food-borne variant due to the ingestion of improperly stored food is prevalent [1]. The classic clinical picture is symmetrical palsy that can vary from mild oculopharyngeal-facial deficits to the neuroparalytic flaccid syndrome affecting the cranial nerves, limbs and respiratory muscles with autonomic failure [2]. Here we described an atypical presentation of food-borne type B botulism with a benign clinical course characterized initially by unilateral tonic mydriasis.

## Case presentation

**Fig. 1:**
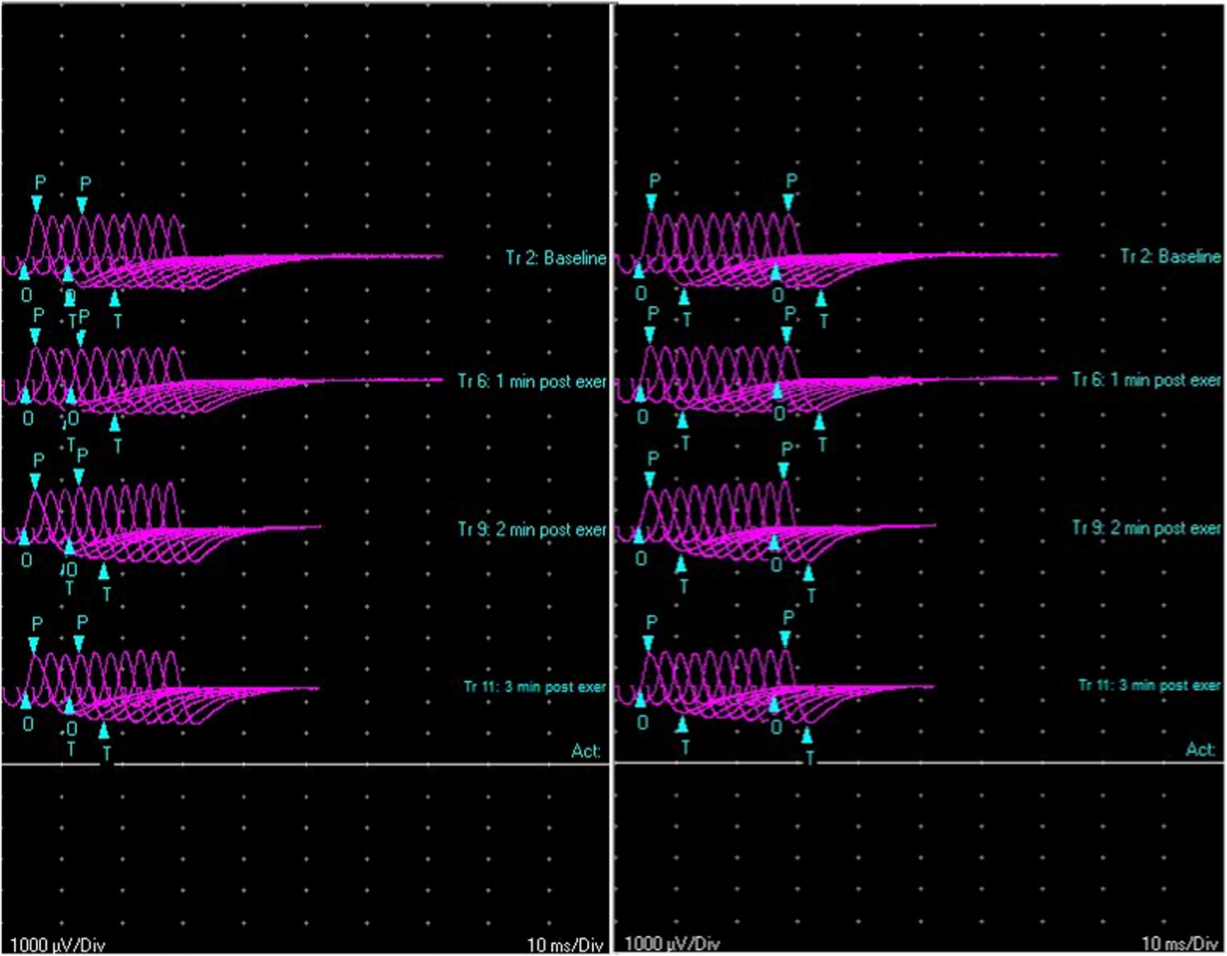
Post-tetanic facilitation in response to high frequency (20-Hz) RNS recording from orbicular nasalis muscle during the first two minutes of stimulation after exercise. The slight (18%) amplitude increase of the 4th CMAP compared to the 1st CMAP in the left panel. The most significant (30%) increase of the 10th CMAP compared to the 1st CMAP in the right panel. The response pattern at high frequency RNS resembles that found in Lambert-Eaton syndrome, which, unlike botulism, shows a more evident anomaly (increase in CMAP amplitude > 100%)

**Fig. 2:**
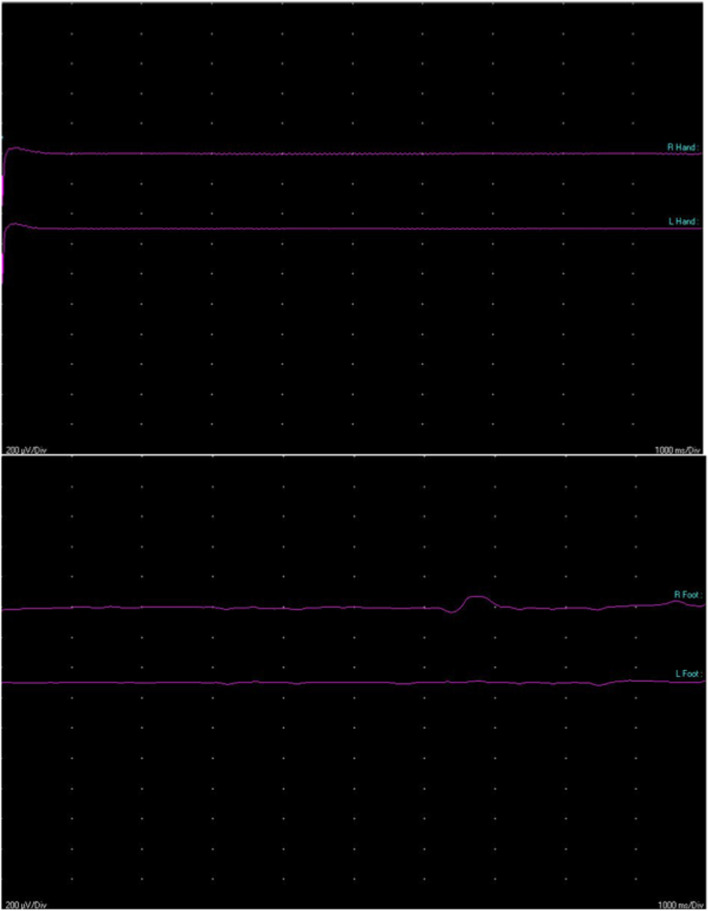
Absent sympathetic skin response was recorded from both left and right hands (panel above) and feet (panel below). Technically, the surface electrodes were placed on the palm and dorsum of the hands and feet. Stimuli are delivered at the left median nerve at an irregular interval time. Each stimulus consisted of a single electric pulse of 0.5 ms duration and intensity set to be 1.5 higher than the motor threshold of the stimulated nerve. The response was considered absent because no consistent voltage change was detected. This finding indicates the generalized sudomotor function impairment

A 26-years old man with a past medical history of hyperthyroidism under treatment presented at the Emergency Department (ED) complaining of blurred vision. He lived alone, no fever or flu-like syndrome was reported in the previous days. The first neurological and ophthalmologic examination revealed a mydriatic (pupil size 8 mm) right pupil unresponsive to light and near. The pharmacological eye drops test resulted in constriction of the dilated pupil after concentrated pilocarpine installation; no other sensorimotor abnormalities in cranial nerves, limbs and trunk were observed; the fundoscopic inspection did not demonstrate pathologic findings; the blood cell count and thyroid function test were within normal limits. He was first suspected of a brainstem cerebrovascular accident or symptomatic intracranial vascular anomaly, but brain/neck computed tomography (CT) angiography and non-contrast-enhanced brain magnetic resonance imaging (MRI) were negative. Subsequently, inflammatory and neuroinfectious aetiologies were ruled out through cerebrospinal fluid analysis. The patient was discharged with a presumptive diagnosis of benign episodic unilateral mydriasis and the indication for an outpatient follow-up visit. After 24h, the patient returned to the ED because of worsening blurred vision. The neurological re-examination revealed bilateral asymmetric mydriasis (right pupil size 9 mm, left pupil size 5 mm) and pupils that fail to constrict in response both to light and accommodation. The repeated blood analyses were regular, serological screening tests for common infective or rheumatologic disease were negative, and the contrast-enhanced MRI of the brain, orbit and spinal cord were consistently unremarkable. Two days later, the patient began to suffer from fatigue and mild constipation. Nerve conduction studies showed normal conduction velocities, latency, and amplitudes in the limbs' main motor and sensory nerves. The needle electromyography (EMG) and both 3-and 20-Hertz (Hz) repetitive nerve stimulation (RNS) of limbs’ muscles were within limits. Recording from orbicular nasalis muscles, only RNS at the frequency of 20-Hz showed a slight (18%) and wider (30%) increment in compound muscle action potential (CMAP) amplitude, respectively, in the fourth compared to the first and in the tenth compared to the first potential, both intended as phenomena of facilitation, in the absence of post-tetanic exhaustion (Fig. 1). The neurophysiological examination was completed by sympathetic skin response (SSR), which resulted in its absence in the hands and feet (Fig. 2). The single-fiber electromyography (SFEMG) was not concluded because the patient did not tolerate it. In light of these electrodiagnostic findings, possible botulism was diagnosed. After a more detailed anamnestic collection, it emerged that the patient had eaten once time nine days prior pasta topped with homemade-vegetable sauce (food that, if improperly processed, can provide the right conditions for the growth of C. botulinum*)*. According to the national current regulation, the suspicion of the disease was notified to health authorities. Considering the absence of generalized weakness, dyspnea or dysphagia at the time of the last examination, the indication for the antitoxin therapy was not given. The lactulose syrup was the only treatment administered on-demand for constipation. The stool- and rectal swabs specimens collected in the following two days were sent to a specialized public health laboratory for confirmation of food-borne botulism. The real-time polymerase chain reaction analysis was positive for identifying the gene coding for C. botulinum type B toxin. During hospitalization, prolonged ECG and blood pressure monitoring resulted within normal limits, and the patient started showing spontaneous improvement sixteen days after ingesting contaminated food.

## Discussion and conclusions

Botulism can manifest across a broad spectrum of signs and symptoms, and asymmetric distribution, mainly involving the voluntary musculature, can occur in 7% of the case [2], which make it prone to misdiagnosis, especially if a patient is encountered outside of an outbreak.

Clinical variability may depend on the pattern of exposure to the BoNT (e.g. in the wound or iatrogenic botulism, the symptoms appear early and remain more evident in those voluntary muscles in contact with the toxin first), or on a particular intrinsic susceptibility of some parts of the body to it, such as that of the oculomotor nerves. The absence of signs (e.g. ptosis, strabismus) or symptoms (e.g. diplopia) of external ocular muscles palsies is atypical in botulism [3]. Fixed dilated pupils and subsequent blurred vision as consequence of presynaptic inhibition of ACh release from the parasympathetic component of the short ciliary nerves innervating the iris sphincter muscles [4], is a common symptom of food-borne botulism and, during poisoning of BoNT/B, it can occur in the absence of other motor deficit and cranial nerve abnormalities [5]. It is possible a prominent vulnerability of the autonomic system to C. botulinum toxin type B with respect to the voluntary neuromuscular junction [6]. The pupillary involvement in the food-borne botulism scenario is expected to be typically bilateral, synchronous and symmetric, as recently described in a published report on the clinical features of more than 300 botulism cases collected in the U.S.A. over about ten years (2). In this aspect, the particularity of our case for the unilateral onset and the asymmetrical evolution of the sole ocular autonomic dysfunction. Complete bilateral symmetric internal ophthalmoplegia was reported as exclusive manifestation of food-borne botulism [7]. To our knowledge, only two other reports on lateralized tonic mydriatic pupil as the onset of food-borne botulism are described, which differs from our for the worse clinical course with the bulbar [8] and multifocal autonomic and voluntary motor ocular-orofacial deficit [9]. In clinically atypical and mild cases of food-borne botulism, the definition of an early neurophysiological process can be complex because the disease is not only intuitively tricky to recognize but is generally a disease with no single electrodiagnostic finding specific to it. The small amplitude of the CMAP at rest, the post-exercise facilitation with RNS and the increase in jitter shown by SFEMG are considered consistent in diagnosing presynaptic neuromuscular junction disorders. However, they are limited by their broad variability depending on clinical severity, the need for protracted patient collaboration, and not providing information on autonomic dysfunction [10]. The initial positive response to the concentrated pilocarpine eye drops test, the incremental response < 100% at the high rate RNS at the facial level, and the absent SSR in a patient with prominent autonomic neuro-ophthalmological signs allowed us to suspect the disease. Two types of responses in the needle EMG and RNS are possible in botulism, depending on the disease's clinical course. In the severe course, a small CMAP amplitude at rest, a decremental response at a low rate RNS and a negligible incremental response at a high rate RNS are observed; in the benign course, normal CMAP amplitude at rest, no change in response to a low rate RNS, and a significant incremental response at a high rate RNS occur. An explanation for this difference is that in the severe form of botulism, the presynaptic block may be so diffuse and grave in the nerve terminals that the high stimulation rate fails to release acetylcholine [11]. In addition, the more incremental response of the 10th versus the 1st CMAP to high rate RNS was suggested as an electrodiagnostic diagnostic marker of botulism more specific to that obtain evaluating the response between the 4th and the 1st CMAP [12]. The sympathetic skin response (SSR) is a slow wave resulting from activation of the sympathetic postganglionic fibres innervating sweat glands. It represents a simple, effective method to evaluate autonomic functions in the daily clinical context. Only its absence, in the appropriate clinical scenarios, is considered pathological. We reported the SSR absent in the four limbs of the patient without him suffering from anhidrosis. This is not surprising as no linear relationship has never been demonstrated between SSR findings and the symptoms of botulism [13]. This report highlights that botulism should always be suspected when faced with a case of asymmetric (or unilateral) internal ophthalmoplegia. Moving to an exhaustive anamnesis and appropriate neurophysiological evaluation is critical in the diagnostic work-up. Pharmacologic eye(s) drop testing of the dilated pupil(s) with varying concentrations of pilocarpine can demonstrate constriction of the tonic dilated pupil(s) following 1–2% concentrated pilocarpine instillation. Thereafter, the RNS (with particular attention to stimulating at least one muscle of the most affected parts of the body) and SSR are useful for botulism diagnosis, even in the cases without signs of weakness and where mild generalized autonomic dysfunction may be underestimated without appropriate testing.

## Data Availability

Data sharing is not applicable to this article as no datasets were generated or analyzed during the current study. The authors are available to answer the questions related to this case report.

## Abbreviations

ACh: Acetilcholine
BoNT: Botulinum neurotoxin
ED: Emergency Department
CT: Computed Tomography
MRI: Magnetic Resonance Imaging
ms: Millisecond
EMG: Electromyography
RNS: Ripetitive Nerve Stimulation
Hz: Hertz
CMAP: Compound motor action potential
SSR: Sympathetic Skin Response
